# Adoption and feeding of fieldfare nestlings and fledglings by European blackbird

**DOI:** 10.1002/ece3.10169

**Published:** 2023-06-15

**Authors:** Dariusz Wysocki, Katarzyna Wawryniuk, Karolina Cieślińska, Katarzyna Wojczulanis‐Jakubas

**Affiliations:** ^1^ Institute of Marine & Environmental Sciences University of Szczecin Szczecin Poland; ^2^ Department of Vertebrate Ecology and Zoology, Faculty of Biology University of Gdańsk Gdańsk Poland

**Keywords:** alloparental care, cross‐feeding, interspecific interactions

## Abstract

Interspecific adoption is an intriguing topic in behavioral and evolutionary ecology. As it is a rare phenomenon, seldom documented in the literature, reports of interspecific adoption based on solid data are particularly valuable. A long‐term and extensive monitoring programme involving a local population of European blackbirds (*Turdus merula*, hereafter blackbird) has yielded, among other things, observations of alloparental behavior exhibited by blackbirds toward fieldfare (*Turdus pilaris*) nestlings (a single nest, the first‐ever record) and fledglings (12 cases in all). We discuss the observations in the context of the available literature.

## Graphical abstract

Here, we describe observations of alloparental behavior exhibited by blackbirds toward fieldfare (*Turdus pilaris*) nestlings (single nest, first record ever) and fledglings (12 cases in total). We discuss the observations with respect to available literature.

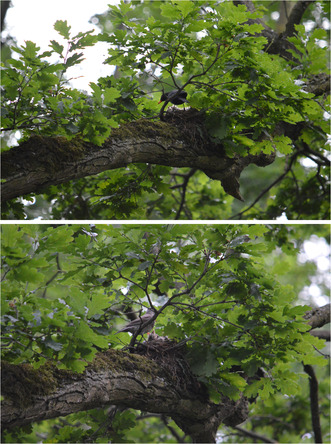

## 1 INTRODUCTION

With parental care being costly (Clutton‐Brock & Vincent, 1991), alloparental behavior is an interesting issue in an evolutionary sense, as benefits to the step‐parents may not be obvious (Kalmbach, 2006). Nevertheless, the phenomenon has been reported across various animal taxa and actually appears to be quite widespread, especially among birds (Brown, 1998; Bukaciński et al., 2000; Pierotti, 1991; Riedman, 1982). While the majority of reports relate to intraspecific interactions (Avital et al., 1998; Brown, 1998; Bukaciński et al., 2000; Castillo‐Guerrero et al., 2014; Pierotti, 1991; Riedman, 1982; Wysocki et al., 2017), where the birds' behavior could be adaptive (various hypotheses, as reviewed in Kalmbach, 2006), there are few reports of interspecific adoption (Moore, 1973, Shy, 1982). In this case, the adaptive significance is doubtful, and the most commonly evoked Reproductive Error Hypothesis explains such an adoption through the perspective of parental mistakes in the recognition of their young (Andersson & Eriksson, 1982; Riedman, 1982). However, given the scarcity of reports on interspecific adoption in the literature, drawing any inferences whatsoever about the mechanisms and phylogenetic distribution of such interspecific adoption must be premature. This underlines the need to report such behavior, on condition that it can be reliably recorded.

Here, on the basis of an extensive, long‐term monitoring programme involving a local population of the European blackbird (*Turdus merula*, hereafter blackbird), we describe observations of alloparental behavior exhibited by blackbirds toward fieldfare (*Turdus pilaris*) nestlings (the first‐ever record) and fledglings (11 cases of adoption).

## 2 STUDY AREA AND METHODS

The studied population of blackbirds is situated in two urban parks in Szczecin (NW Poland; for a detailed description of the study area, see Wysocki, 2004). Every year since 2000, over 90% of this population has been color‐ringed during the breeding season (March–August). The nests of the ringed birds are located by daily observations, then tracked every 2–3 days until fledging (for more details on the fieldwork, see Cholewa et al., 2021). Fieldfares also breed in the study area: ca 5% of the breeding population are color‐ringed annually, but no specific studies have been undertaken on this species.

During the 26 years of this fieldwork, we recorded fieldfare nestlings and fledglings being fed by adult blackbirds. However, we never observed the opposite situation, that is, blackbird fledglings being fed by fieldfares. When an adult blackbird fed a fieldfare fledgling at least twice during at least 1 day, we assumed this behavior to be a sign of adoption; we did not treat purely begging behavior by the fledglings directed toward adults (sometimes observed in the study area) as adoption.

## 3 RESULTS

### 3.1 Adoption of fledglings

We observed 11 cases of interspecific adoption (expressed as multiple feeding events), all of them being perceived as the adoption of one or two fieldfare fledglings by a blackbird, never the other way around (Table 1). From 1997 to 2022, we recorded 2905 broods of blackbirds (1008 successful). Interspecific adoption occurred four times following nest predation and six times after a successful breeding attempt. The probability of interspecific adoption after nest predation is, thus, 0.2% and after successful breeding 0.6% (for approximately 60 pairs of blackbirds and 30 pairs of fieldfare on the 24 ha area of the city park). We never observed the same individual adopting fieldfares more than once. Both blackbird sexes were involved to an equal extent in adopting the fieldfare fledglings (5 females and 6 males); moreover, no apparent pattern could be discerned in the age structure of the adopting parents (females from 3 to 6 years old and males from 3 to 7 years old; Table 1). In four cases, the adopting parents had lost their own brood, in six cases, their own offspring was alive and still regularly fed by the partner, without the adopter's contribution, and in one case the previous history of adopting parent was not known (Table 1). After the adoption event, the majority of the adopting parents (80%) did not undertake a further breeding attempt in the given season (Table 1).

**TABLE 1:** Details of records of the adoption of fieldfare fledglings and nestlings by adult blackbirds.

Time of the adoption event	Number of adopted fledglings/nestlings	Characteristics of the adopting parent		Breeding history of the adopting parent (own nestlings/fledglings) with respect to the adoption event	
		Sex	Age (years)	Before/during	After
20 June 1997	1	Female	3	Nest predated 3 days before the adoption	One further brood after the adoption
23–27 May 2002	1	Male	3	Only the male was feeding the adopted fledgling (his own fledglings were 18 days old at the time when the adoption event was first noticed. His mate was feeding their own four fledglings)	No brood after the adoption
15 June 2002	2	Male	3	Unknown	No brood after the adoption
1–17 June 2003	1	Male	7	Nest predated 2 days before the adoption	No brood after the adoption
27–29 May 2004	1	Male	2	The male was feeding the adopted fledgling and two of his own ones (the latter were 21 days old at the time when the adoption event was first noticed). His female partner was feeding their own single offspring	No brood after the adoption
21 June–5 July 2005	2	Male	5	The male was feeding the adopted fledgling and two of his own ones (his own fledglings were 21 days old at the time when the adoption event was first noticed). His female partner was feeding only one of their own fledglings	No brood after the adoption
25 June 2006	1	Male	6	Nest predated 4 days before the adoption	No brood after the adoption
24 July 2008	2	Female	4	The female was feeding two adopted fledglings and her own 21‐day‐ old single fledgling. Her male partner was absent	No brood after the adoption
24–26 June 2014	1	Female	4	Nest predated 3 days before the adoption	No brood after the adoption
18–29 July 2019	2	Female	4	The female was feeding only the two adopted fledglings. Her male partner was feeding their own three 18‐day‐old fledglings	No brood after the adoption
19–22 May 2021	1	Female	6	The female was feeding only the adopted fledgling. Her male partner was feeding their own four 19‐day‐old fledglings	One brood more after adoption
16 June–2 July 2022	5	Male	7	The female was feeding her own nestlings alone; the male was feeding the fieldfare nestlings and continued to do so after they had fledged	No brood after the adoption

### 3.2 Nestling adoption

**FIGURE 1:**
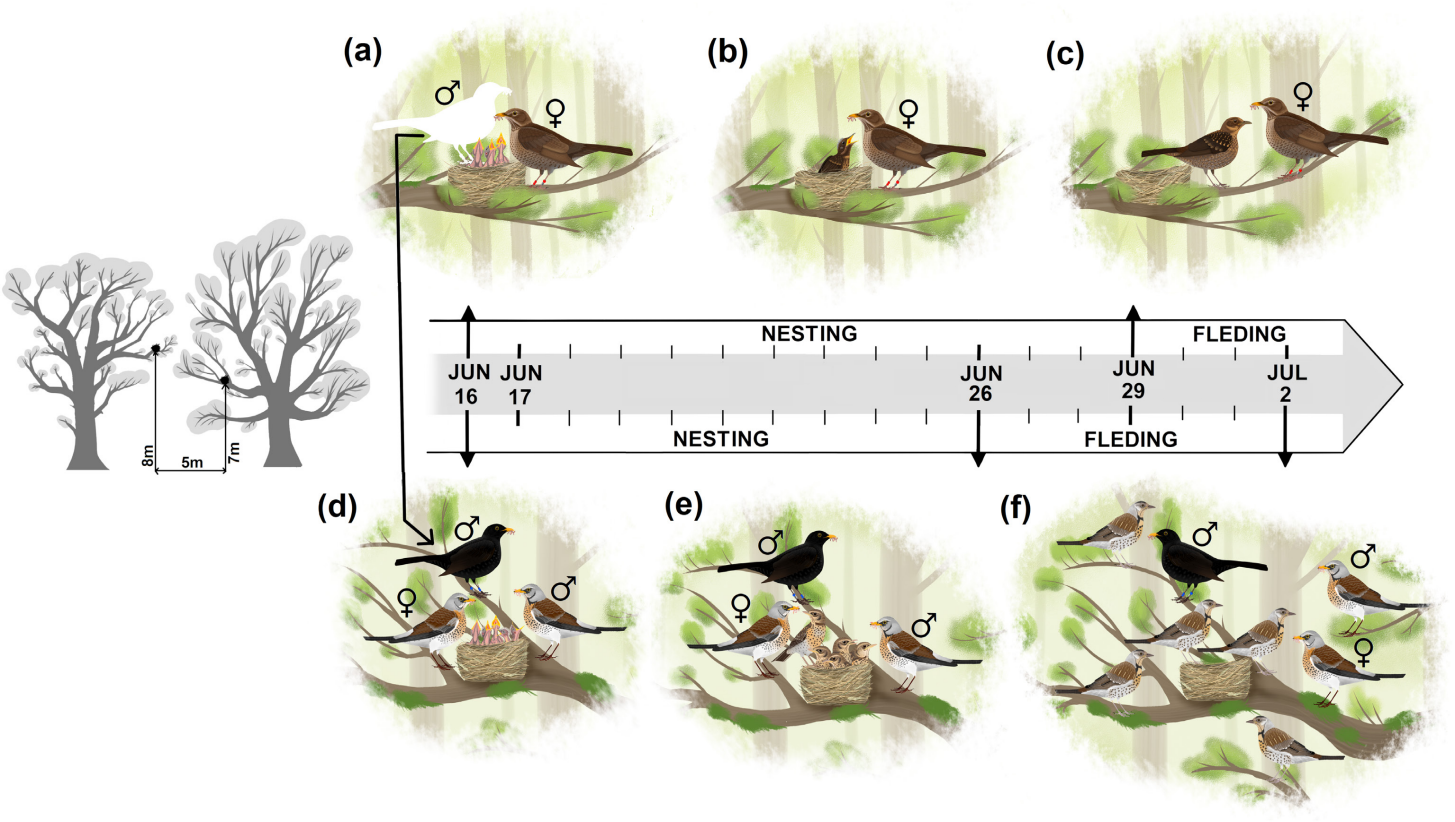
The history of fieldfare nestlings adopted by a male blackbird, with the nest of the blackbird shown in the upper panel, that of the fieldfare in the lower panel: (a) and (d) the male blackbird is seen for the first time feeding the fieldfare nestlings but not his own; at the same time the female blackbird and the two fieldfare parents are observed feeding their own offspring; (b) and (e) the male blackbird is seen continuing to feed the fieldfare nestlings but not his own; the female blackbird and the two fieldfare parents are still feeding their own offspring, but the size of the blackbird brood has been reduced by predation (not shown in the illustration); (c) and (f) the male blackbird is seen feeding the fieldfare fledglings but not his own; the female blackbird and the two fieldfare parents are observed feeding their own fledglings.

Although there were previous observations of blackbirds feeding fieldfare fledglings, the first event of a blackbird feeding fieldfare nestlings (five individuals) was observed on 16 June 2022 (Figure 1). The fieldfare chicks were 5 days old on that day. The fieldfare nest was then monitored from 17 to 23 June (until the chicks fledged), and the male blackbird continued the feeding throughout these observations (a total of 5 h of observations split into 10 min intervals). The last feeding of the young fieldfares (already fledglings) by the male blackbird was seen on 2 July (9 days after fledging). Throughout the observations, we could see that both fieldfare parents were provisioning their nestlings, and on two occasions the male blackbird was seen chasing the parents away. The color rings on the male blackbird enabled us to identify him, that is, the same individual throughout the observations and to establish his breeding history in that season. We, therefore, know that at the time of the alloparental feedings by the male blackbird, his partner (also color‐ringed) fed their chicks by herself until they fledged (29 July, based on 4 h of observations of the blackbird nest). The male blackbird was never seen feeding his own nestlings and fledglings. There were three blackbird chicks (2 days old) in the nest on 16 June but probably only one of them survived to the age of 14 days (the usual age when blackbirds fledge). We know that the nest was predated on 22 June, when one nestling was killed but that the two other survived, at least until the next day. The nests of the focal blackbirds and fieldfares were 5 m apart from each other, both situated on a branch of an oak tree, at 8 and 7 m above the ground, respectively.

**FIGURE 2:**
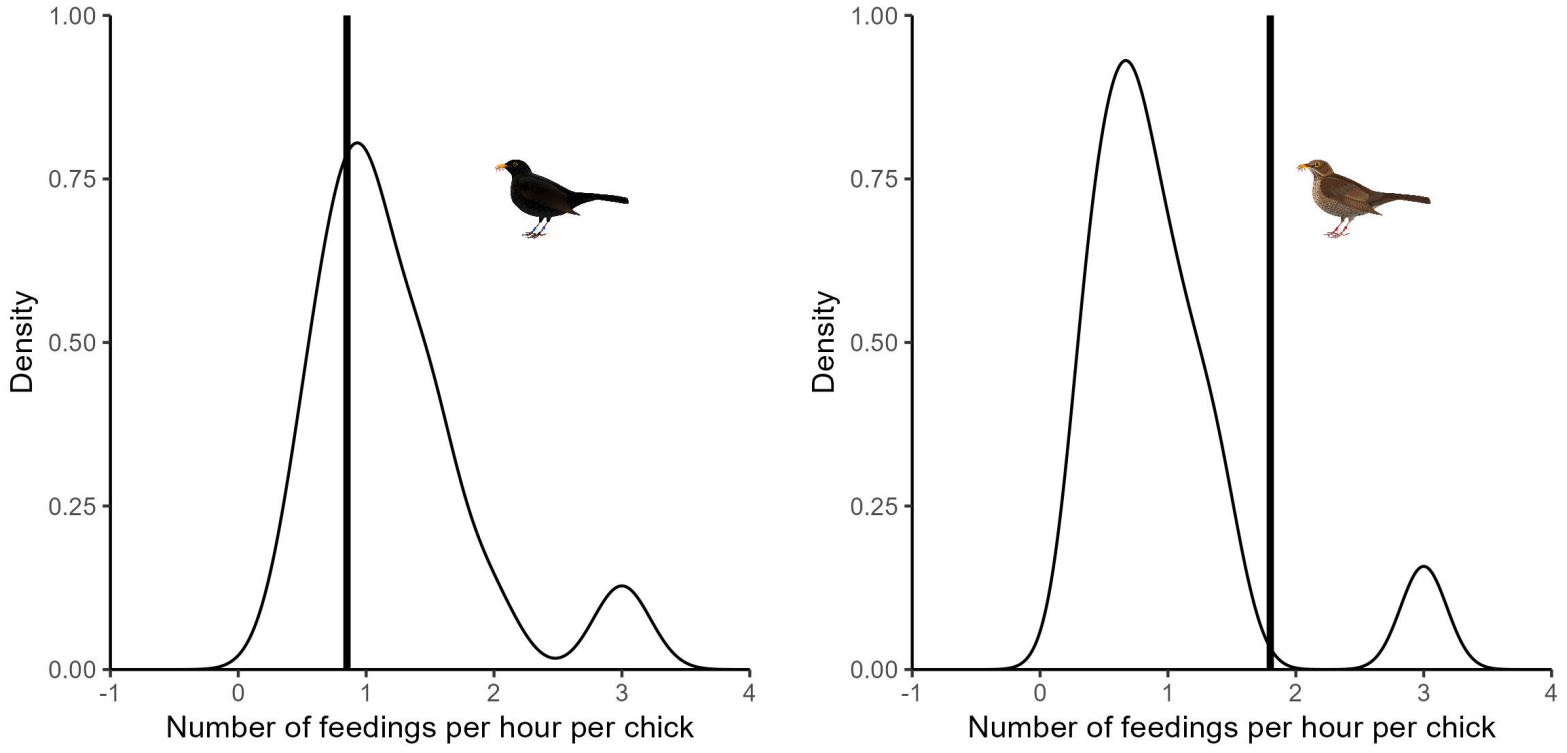
Distribution of feeding rates of male blackbirds (left‐hand panel) and female blackbirds (right‐hand panel; number of feedings per nestling per hour) on day 6–8 of a chick's life; based on data from 42 nests in an urban population (Szczecin, Poland). The vertical lines denote the feeding rates by the male blackbird that had apparently abandoned his own brood to provide alloparental care, and by the female blackbird deserted by the male.

During all the observations of both the blackbird and the fieldfare nests, we recorded the number of feedings performed in the nest by each identifiable parent and calculated the feeding rate, that is, the number of feedings performed by the parent per chick per hour. Thus, we recorded the feedings of the fieldfare nestlings by the male blackbird (0.8 feedings/nestling/h), the feedings of the blackbird nestlings by the female blackbird (1.8 feedings/nestling/h), and the feedings of the fieldfare nestlings by the two fieldfare parents (1.8 feedings by both parents/nestling/h, that is, 0.9 feedings/nesting/h by one parent). The feeding rate of the focal male blackbird was well within the range of the values exhibited by randomly sampled male blackbirds from the same population (*p* = .33; tested in a bootstrap procedure with 10,000 iterations; Figure 2). The feeding rate of the focal female blackbird was high compared with the overall feeding rate of female blackbirds in the study population; only 7% of female blackbirds randomly sampled from the same population exhibited higher values (*p* = .07; tested in a bootstrap procedure with 10,000 iterations; Figure 2).

## 4 DISCUSSION

We report observations of fieldfare nestlings and fledglings being fed by adult blackbirds. The observations of nestling feeding are from a single nest here, but to the best of our knowledge, it is the first such record for either species. It is also a true nestling adoption, as it resulted in the adopting male abandoning his own nest, and all his feeding effort appeared to be directed to the adopted offspring (Figure 2). At the same time, his female partner increased the feeding rate in their nest, possibly to compensate for the missing contribution of the male (Figure 2). The feeding of fieldfare fledglings by blackbirds seems to be much more common, as here we report 11 cases [we mentioned about seven of them without any detailed description in our earlier study on conspecific fledgling adoption in blackbirds (Wysocki et al., 2017)].

No matter how extraordinary cross‐species adoption or feeding is, blackbirds are among the species that seem most often to feed the young of other species (as reviewed in Shy, 1982). As regards the nestling stage, a male blackbird assisting a female song thrush (*Turdus philomelos*) in feeding her two nestlings (Moore, 1973) has been reported. With regard to fledglings, there have been reports on at least three other species: Pied Wagtail, Robin, and Jay. Perhaps one of the most unusual cases is described by Lack (1953), when a female Blackbird, having reared her own young, continued to offer food to any bird coming near for 2–3 weeks. Probably the most unusual observation is that of a blackbird feeding a young jay (*Garrulus glandarius*). This was not reported in the scientific literature but described at (https://www.bto.org/our‐science/projects/gbw/gardens‐wildlife/garden‐birds/behaviour/cross_species_feeding). Jays are considered predators of blackbirds (on eggs and young).

Without more extensive data, it is impossible to perform a meaningful analysis to explore the possible cause(s) of the observed adoptions, and one can only speculate about the mechanisms involved. The reproduction error hypothesis, where parents simply mistake their offspring (Andersson & Eriksson, 1982; Riedman, 1982), seems to be the most reasonable in all the reported cases. The two species co‐occur in the study area, breed at roughly the same time, and their offspring, until a certain age, are similar in size and appearance. All this may facilitate parental behavior being focused on the wrong birds. This could be a problem, particularly during the fledging period, when the young birds are no longer associated with a fixed nest site and roam over a wider area. However, such a mistake can easily occur during the nesting period, too, when nests of the two species are close to each other (here: 5 m apart), and acoustic stimuli from one (foster) nest are particularly strong (perhaps stronger than in the other; five 5‐day‐old fieldfare nestlings vs. three 2‐day‐old blackbird nestlings). Unidirectional adoption, that is, adult blackbirds adopting fieldfares but never the other way round makes the reproduction error hypothesis even more likely. This is because the fieldfare is often a colonially nesting species (Cramp & Simmons, 1980), whereas the blackbird is a solitary breeder. Then, according to Bustamante and Hiraldo (1993), colonially breeding species may be better at recognizing their offspring than solitaries, because fledgling intrusions are potentially more frequent in a colonial context.

Another reason for cross‐feeding/adoption could be the hormonal priming of the parents (Jouventin et al., 1995). In all the cases where a blackbird's own brood was predated or it had only a small number of its own fledglings, adults of both sexes in which prolactin (parenting hormone) levels were still high, could have fed whatever fledgling was close by. Given the fact that the prolactin level is known to depend on stimuli from the eggs or the chicks, parents with high concentrations of the hormone could also have been more responsive to any stimuli from the chicks (Angelier et al., 2016). This might be further reinforced by directed begging behavior of fieldfare fledglings toward any parent that could provide food (Shy, 1982).

Both male and female blackbirds of different ages were involved in the adoption of young fieldfares. In our earlier study on conspecific fledgling adoption in the blackbird, young males were found to adopt more frequently than older males or females (Wysocki et al., 2017). It was then suggested that such adopting behavior in young males might enable them to gain experience for future breeding (Komdeur, 1996; Simmons, 1992) and that this could be more important for males than for females (Jarska et al., 2015; Wysocki, 2004, 2006). Alternatively, young males, being inexperienced, are more likely to make a mistake in recognizing their offspring. The observations of cross‐species adoption given here do not suggest such a pattern, but it would be premature to draw any specific conclusions, given the modest sample size.

Finally, it is worth highlighting the fact that these observations are an effect of a long‐term research monitoring programme of a local population. Long‐term studies are not common (for various reasons), but they are very important for acquiring an integrated understanding of the study system (e.g. Lindenmayer et al., 2012). Importantly, they also provide an opportunity to observe various behaviors that are not so frequent and, thus, hard to investigate in a short‐term study. Moreover, such observations, although made opportunistically but associated with ongoing long‐term research, can be described with various important details, which might otherwise be simply lost.

## AUTHOR CONTRIBUTIONS

**Dariusz Wysocki:** Conceptualization (lead); data curation (lead); formal analysis (supporting); investigation (equal); methodology (lead); supervision (lead); writing – original draft (equal). **Katarzyna Wawryniuk:** Formal analysis (supporting); investigation (lead); writing – original draft (supporting). **Katarzyna Wojczulanis‐Jakubas:** Conceptualization (supporting); formal analysis (lead); writing – original draft (equal). **Karolina Cieślińska:** Visualization (lead); writing – original draft (supporting).

## ACKNOWLEDGEMENTS

The authors are grateful to all the students and volunteers for their help with the fieldwork. We would like to thank Peter Senn and the anonymous referee for improving the language.

## FUNDING INFORMATION

None.

## Data Availability

All data are included in Figshare: doi: 10.6084/m9.figshare.23212994
